# Prospects in Folate Receptor-Targeted Radionuclide Therapy

**DOI:** 10.3389/fonc.2013.00249

**Published:** 2013-09-24

**Authors:** Cristina Müller, Roger Schibli

**Affiliations:** ^1^Center for Radiopharmaceutical Sciences ETH-PSI-USZ, Paul Scherrer Institute, Villigen-PSI, Switzerland; ^2^Department of Chemistry and Applied Biosciences, ETH Zurich, Zurich, Switzerland

**Keywords:** folate receptor, folic acid, radionuclide therapy, cancer

## Abstract

Targeted radionuclide therapy is based on systemic application of particle-emitting radiopharmaceuticals which are directed toward a specific tumor-associated target. Accumulation of the radiopharmaceutical in targeted cancer cells results in high doses of absorbed radiation energy whereas toxicity to non-targeted healthy tissue is limited. This strategy has found widespread application in the palliative treatment of neuroendocrine tumors using somatostatin-based radiopeptides. The folate receptor (FR) has been identified as a target associated with a variety of frequent tumor types (e.g., ovarian, lung, brain, renal, and colorectal cancer). In healthy organs and tissue FR-expression is restricted to only a few sites such as for instance the kidneys. This demonstrates why FR-targeting is an attractive strategy for the development of new therapy concepts. Due to its high FR-binding affinity (*K*_D_ < 10^−9^ M) the vitamin folic acid has emerged as an almost ideal targeting agent. Therefore, a variety of folic acid radioconjugates for nuclear imaging have been developed. However, in spite of the large number of cancer patients who could benefit of a folate-based radionuclide therapy, a therapeutic concept with folate radioconjugates has not yet been envisaged for clinical application. The reason is the generally high accumulation of folate radioconjugates in the kidneys where emission of particle-radiation may result in damage to the renal tissue. Therefore, the design of more sophisticated folate radioconjugates providing improved tissue distribution profiles are needed. This review article summarizes recent developments with regard to a therapeutic application of folate radioconjugates. A new construct of a folate radioconjugate and an application protocol which makes use of a pharmacological interaction allowed the first preclinical therapy experiments with radiofolates. These results raise hope for future application of such new concepts also in the clinic.

## Folate Receptor and Folic Acid

Folate receptors (FRs) are glycoproteins with molecular weights of 38–44 kDa which exist in several isoforms (1, 2). The FR-α and the FR-β are both anchored in the cell membrane by a glycosyl-phosphatidyl-inositol domain. The FR-α is expressed at a few sites of normal epithelial membranes for instance in the proximal tubule cells of the kidneys (3–5) and in a variety of solid cancer types of epithelial origin (6). Cancer types with the highest frequency of FR-α expression are ovarian (90%), endometrial (90%), brain (90%), lung (78%), and renal carcinomas (75%) (6–9). On the other hand, FR-expression in cancer of the head and neck (52%), breast (48%), stomach (38%), and colon-rectum (32%) was found at intermediate frequencies (6, 9). It is important to recognize that the expression level of the FR-α on tumors may have a prognostic value as it has been found to correlate with the survival rate of the patients. In ovarian, endometrial and breast carcinomas as well as in primary and metastatic colorectal cancer overexpression of the FR-α correlated with a worse survival (10–13). In contrast, evaluation of a large number of non-small cell lung cancers revealed that high FR-expression levels correlated with a better survival (14).

The FR-β is found in the placenta and in normal hematopoietic tissue (e.g., spleen, thymus, and monocytes) (15, 16) but also in hematopoietic malignancies such as acute and chronic myelogenous leukemia (17) and on activated macrophages (16). Targeting of the FR-β expressed on activated macrophages has initially been associated primarily with targeting of inflammatory diseases such as rheumatoid arthritis (9, 18, 19). However, the FR-β may have future implications also in the field of oncology as a marker of tumor-associated macrophages (20, 21).

The vitamin folic acid binds with high affinity to the FR (*K*_D_ = 10^−9^ M) and hence, it can be used as a targeting ligand. Compared to other (e.g., peptide- or protein-based) targeting agents the use of folic acid provides several advantages (22). Folic acid is small in size (441 Da) and accessible for chemical modification. Moreover it is relatively stable in solution of a wide range of pH-values and at elevated temperatures (22). Due to its function as a vitamin, folic acid is not toxic to healthy organs and tissue nor does it provoke immune reactions. Since binding to the FR results in an endocytotic uptake of folic acid cellular delivery of even larger payloads which are conjugated to folic acid is accessible (23, 24). All of these features together make folic acid an excellent candidate for tumor targeting allowing selective delivery of attached probes to FR-expressing (cancer) cells.

## FR-Targeted Therapies

In the first instance it is the FR-α which attracted most interest as a tumor-associated target for imaging purposes and targeted therapy concepts (9, 25). Targeting of FR-positive tumor cells *in vitro* and *in vivo* has been exemplified by a number of research groups using folic acid conjugates with a variety of therapeutic probes (25, 26). For FR-targeted therapies several concepts have been developed based on e.g., highly toxic chemotherapeutics, drug-loaded liposomes, nanoparticles, and oligonucleotides which were attached to folic acid [reviewed in (27)]. Among those the most advanced approach is clearly the concept of using folic acid-targeted chemotherapeutics, most importantly EC145, a folate conjugate of a vinca alkaloid, desacetylvinblastine monohydrazide (28). Already in 2009 a phase I clinical study was published reporting on the clinical pharmacokinetics and exposure-toxicity relationship of EC145 in cancer patients (29). Meanwhile, several clinical studies performed with EC145 (Vintafolide™) demonstrated the benefit of this therapy for patients with cancer of the lung and ovaries (30–33)

For FR-targeted immunotherapy as another targeted therapy concept folic acid is exploited to carry an attached hapten to the surface of tumor cells (34). The aim of this approach is to render the tumor cells more immunogenic upon FR-binding of the folate-hapten conjugates. The pre-existing or induced immunity of the patient allows the haptens to attract anti-hapten-antibodies and to provoke immune reactions. Hence, it enhances the anticancer immune reaction of the host against hapten-decorated tumor cell (34). Several preclinical studies proved the potential of this concept for cancer therapy (35–37). Another strategy of FR-targeted immunotherapy is the use of FR-binding antibodies (38, 39). Several concepts of (radio)immunotherapy with FR-binding chimeric (e.g., MOv18) and humanized (e.g., MORAb-003) monoclonal antibodies were developed and evaluated in (pre)clinical studies (38, 40–44).

Various different drugs, including chemotherapeutics (e.g., doxorubicin and paclitaxel) have been selectively delivered to FR-expressing cancer cells through use of folic acid conjugated carriers such as liposomes and nanoparticles (26, 45, 46). Possible advantages of this strategy are an increased drug loading capacity and the fact that the larger size of these targeting constructs prevents renal filtration and hence, undesired retention in the kidneys (27). From *in vivo* investigations with FR-targeted carriers labeled with radionuclides (^99m^Tc, ^188^Re, ^68^Ga, ^64^Cu) (47–50) it is apparent that nanoparticles and liposomes usually accumulate in the reticuloendothelial system (RES) while the *in vivo* targeting effect remains poor (47, 48).

## General Features of Folate Radiopharmaceuticals

In the last two decades our group and others have focused on the development of folate-based radiopharmaceuticals (51). The aim was to design new tools for nuclear imaging of FR-positive cancer via single photon emission computed tomography (SPECT) and positron emission tomography (PET) (52–54). More recently, several endeavors were undertaken in view of a therapeutic application of folate radioconjugates using particle-emitting radioisotopes (55–57).

A general feature of folate-based radiopharmaceuticals is their specific accumulation in FR-positive tumor (xeno)grafts. However, accumulation of radioactivity is also always seen in FR-positive organs and tissue such as the kidneys, the salivary glands, and the choroid plexus (58). The distribution profile of radiofolates varied according to their chemical structure with one of the most important feature being a highly hydrophilic character (54). In all of the cases undesired accumulation of radiofolates was found in the renal cortex where the FR is expressed in the proximal tubule cells of the brush border membrane (3, 5, 58). These circumstances resulted in commonly low tumor-to-kidney ratios (<0.2) of radiofolates. This situation is undesired in view of a therapeutic application because of the inherent risk of damage to the radiosensitive kidneys by a high dose burden from particle-radiation (56).

## Strategies to Improve the Tissue Distribution of Radiofolates

A high radioactivity dose burden to the kidneys and as a consequence the risk of damage to the radiosensitive renal tissue particularly in patients with hypertension, diabetes, or other risk factors is an issue in the course of targeted radionuclide therapy (59, 60). Administration of high dosed amino acids, usually lysine and arginine, is a means applied in the clinics to reduce unspecific accumulation of radiopeptides (e.g., somatostatin analogs) in the kidneys (61). However, this strategy is not applicable for folate radioconjugates since other than in the case of radiopeptides, renal accumulation is specific due to binding of radiofolates to FRs (56). Application of diuretics or acidification of the urine did also not have a positive impact on renal retention of radiofolates (56). Therefore, alternative strategies are required. In the first approach antifolates were applied in combination with radiofolates with the aim to improve the tissue distribution profile of radioactivity (62, 63). In a second approach the folate conjugate’s backbone was modified with an albumin-binding entity in order to enhance the circulation time in the blood and therewith possibly increase the tumor-to-kidney ratio (64).

### Application protocol of radiofolates combined with pemetrexed

**Figure 1:**
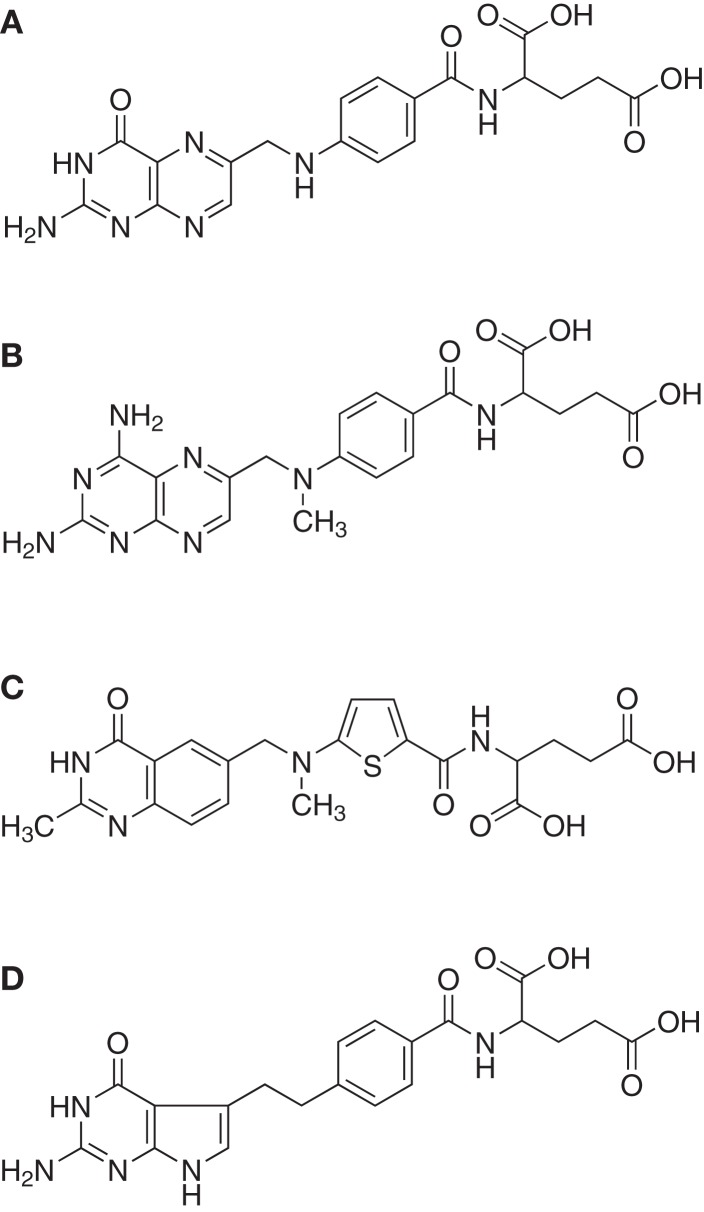
**Folic acid and antifolates**. Chemical structures of **(A)** folic acid and the antifolates **(B)** methotrexate, **(C)** raltitrexed (Tomudex™ ), and **(D)** pemetrexed (Alimta™ ).

The effects of three clinically used antifolates [methotrexate, raltitrexed, and pemetrexed (65), Figure 1] were investigated in combination with a ^99m^Tc(CO)_3_-labeled folate conjugate with the aim to increase the tumor uptake of the radiofolate (62). This study design was based on the observation that incubation of cancer cells with antifolates resulted in an increased uptake of radiofolates *in vitro* (62, 66).

**Figure 2:**
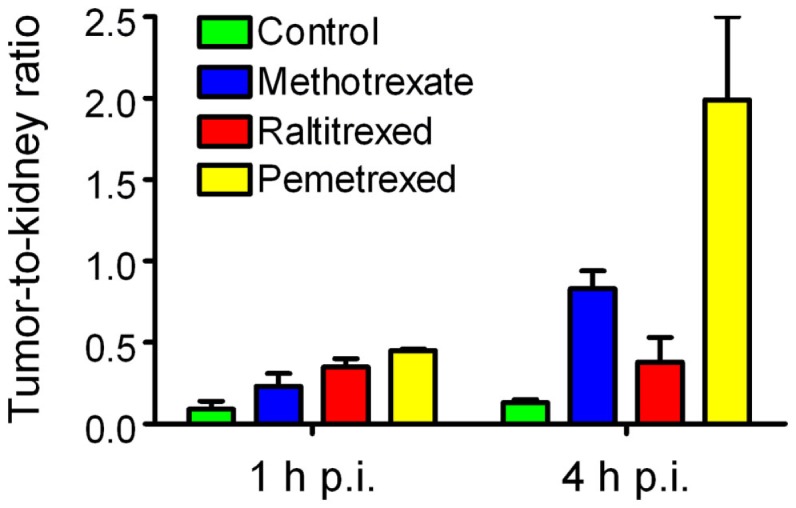
**Effects of antifolates on the tumor-to-kidney ratios of radiofolates**. Tumor-to-kidney ratios obtained at 1 and 4 h after injection of ^99m^Tc(CO)_3_-folate alone (green) or in combination with pre-injected methotrexate (blue), raltitrexed (red), or pemetrexed (yellow).

The *in vivo* data showed that the accumulation of the radiofolate in KB tumor xenografts remained unaffected but a significantly reduced uptake of radioactivity was found in the kidneys. This resulted in clearly improved tumor-to-kidney ratios, with the best results obtained with pemetrexed (Figure 2) (62, 67).

**Figure 3:**
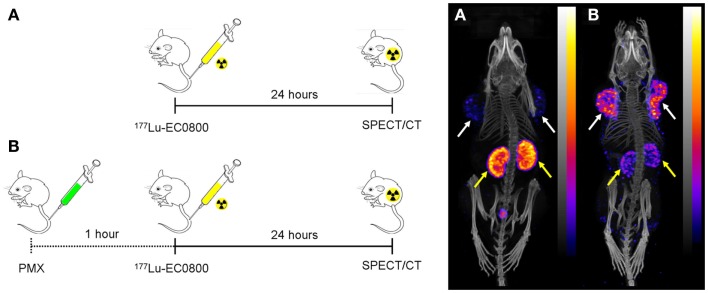
**Effect of pemetrexed on the tissue distribution of radiofolates**. Injection scheme (left) and whole-body SPECT/CT images of KB tumor-bearing mice 24 h after injection of ^177^Lu-EC0800 (right). **(A)** Injection of only ^177^Lu-EC0800 (20 MBq) and corresponding SPECT/CT image. **(B)** Injection of ^177^Lu-EC0800 (20 MBq, Figure 4A) and PMX (0.4 mg) and corresponding SPECT/CT images. Tumors and kidneys are indicated with white and yellow arrows.

The same effect was successfully reproduced in different tumor mouse models (KB, IGROV-1, and SKOV-3 tumors in nude mice and M109 tumors in Balb/c mice) and variable folate radioconjugates [^99m^Tc(CO)_3_-folate, ^99m^Tc-EC20, ^111^In-DTPA-folate, ^111^In/^177^Lu-DOTA-click-folate, ^67^Ga-DOTA-Bz-folate (^67^Ga-EC0800), ^68^Ga-NODAGA-folate] (55, 62, 63, 67–69). The injection protocol and the related SPECT/CT images using ^177^Lu-EC0800 are shown in Figure 3. The underlying mechanism of this effect is not yet clear but a topic of current investigations in our laboratories. The combination of folate radioconjugates and pemetrexed is, however, appealing in view of a therapeutic application. In this respect, we hypothesized that pemetrexed would have a dual role if it was combined with folate-based radionuclide therapy. On one hand it would reduce undesired renal uptake of radiofolates and on the other hand it may have an effect as a chemotherapeutic (70, 71) or radiosensitizing agent (72–74) on the tumor tissue and thus enhance the therapeutic efficacy on the tumors.

### Design of a folate conjugate with an albumin-binding entity

**Figure 4:**
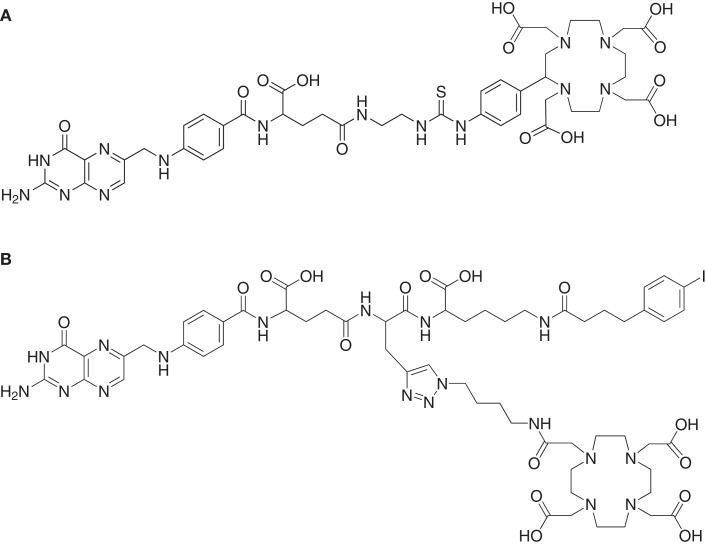
**DOTA-folate conjugates**. Chemical structures of **(A)** EC0800 (56, 64) and **(B)** cm09 (64).

Increasing the serum half-life of pharmaceuticals by serum protein binding may be a measure to improve the pharmacokinetic properties of otherwise rapidly cleared molecules (75). Based on the results which are reported in the literature with antibody fragments that exhibited a non-covalent association with serum proteins (76, 77), it was hypothesized that albumin-binding properties would improve the tissue distribution of radiofolates as well. Therefore, a DOTA-folate conjugate was developed with an integrated small-molecular weight albumin-binding entity which was previously identified from a DNA-encoded chemical library based on the lead structure 4-(*p*-iodophenyl)butyric acid (78). This novel DOTA-folate conjugate (cm09, Figure 4B) was fully evaluated in its ^177^Lu-labeled version and compared with the data obtained with a conventional ^177^Lu-labeled DOTA-folate conjugate (^177^Lu-EC0800, Figure 4A) (64).

**Figure 5:**
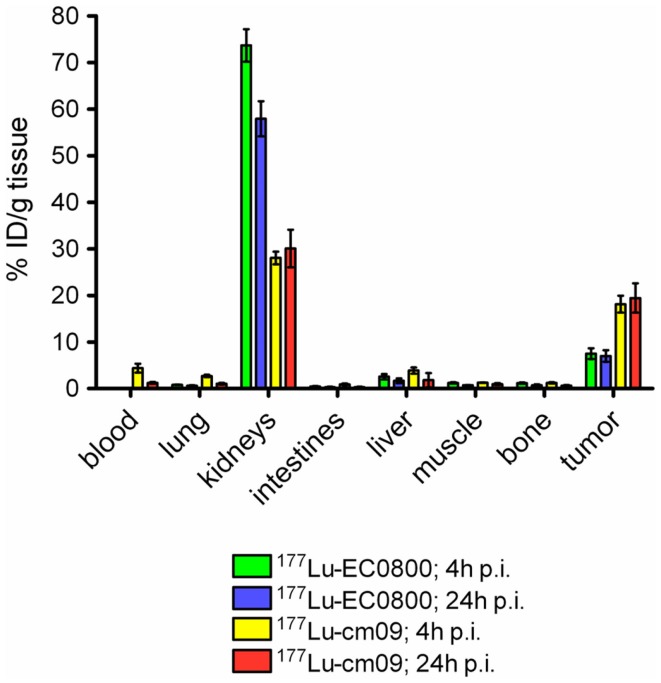
**Comparison of the tissue distribution of ^177^Lu-EC0800 and ^177^Lu-cm09**. Biodistribution data 4 and 24 h after injection of ^177^Lu-EC0800 and ^177^Lu-cm09 in athymic nude mice, bearing KB tumor xenografts.

The *in vitro* cell uptake properties of ^177^Lu-cm09 were comparable to those of other folate radioconjugates (64). Different was however, its feature of binding to plasma proteins as demonstrated by an *in vitro* assay (64). *In vivo* biodistribution studies were performed with ^177^Lu-cm09 and ^177^Lu-EC0800 in athymic nude mice bearing KB tumor xenografts (Figure 5). The enhanced blood circulation time of ^177^Lu-cm09 compared with ^177^Lu-EC0800 resulted in a tumor accumulation of ^177^Lu-cm09 which was ∼2.5-fold higher (∼18% ID/g, 4 h p.i.) than the uptake of ^177^Lu-EC0800 (∼7.5% ID/g, 4 h p.i.) (64). On the other hand, renal retention of ^177^Lu-cm09 was relatively low (∼28% ID/g, 4 h p.i.) compared to other folate conjugates that lack an albumin-binding entity such as ^177^Lu-EC0800 (>70% ID/g, 4 h p.i.) (64). These findings resulted in a sevenfold improved tumor-to-kidney ratio (∼0.7, 4 h p.i.) for ^177^Lu-cm09 compared to the ratio obtained with ^177^Lu-EC0800 (∼0.1, 4 h p.i.). The excellent *in vivo* properties of ^177^Lu-cm09 opened new perspectives for the development of folate-based radionuclide therapy.

To further improve the tumor-to-kidney ratio of the long-circulating ^177^Lu-cm09, biodistribution studies were performed with ^177^Lu-cm09 and pemetrexed using KB tumor-bearing mice. Pemetrexed was administered according to the same application protocol as it was previously employed with conventional folate radioconjugates (55, 63, 67). Independent on whether or not pemetrexed was pre-injected, the tumor uptake was about ∼18% ID/g 4 h after injection. In contrast, reduced renal accumulation (16.81 ± 2.25% ID/g) of radioactivity was found 4 h after injection of ^177^Lu-cm09 if it was combined with pemetrexed compared to the amount of accumulated ^177^Lu-cm09 if it was applied as a single agent (28.05 ± 1.35% ID/g, 4 h p.i.) (64). Thus, pre-injected pemetrexed increased the tumor-to-kidney ratio (1.07 ± 0.25, 4 h p.i) compared to control values at the same time after injection of ^177^Lu-cm09 (0.65 ± 0.07, 4 h p.i.). However, at later time points after injection of ^177^Lu-cm09 the kidney reducing effect of pemetrexed was not observed anymore (64). Most probably, these findings were a result of the fact that pemetrexed was more quickly cleared from the blood circulation than ^177^Lu-cm09. Therefore, the pemetrexed related effect to reduce the kidney uptake of ^177^Lu-cm09 was not maintained for the fraction of ^177^Lu-cm09 which was still in the blood circulation when pemetrexed was already excreted (64).

## Folate Receptor Targeted Radionuclide Therapy – First Results

Both of the aforementioned approaches, a pharmacological intervention with pemetrexed and a chemical modification of the folate conjugate with an albumin-binding entity resulted in improved tissue distribution profiles of radioactivity. Employing either of these approaches for radionuclide therapy in a preclinical setting was the subject of recent research activities in our laboratories. The decay properties of the therapeutic radionuclides which were employed for these studies are listed in Table 1.

**Table 1:** **Therapeutic radioisotopes used for folate-based radionuclide therapy**.

Isotope	^177^Lu	^161^Tb	^149^Tb
Half-life	159.4 h	165.4 h	4.12 h
Eα	–	–	3.967 MeV (16.7%)
E $\beta_{\left(\text{av}\right)}^{-}$	0.134 MeV (100%)	0.154 MeV (100%)	0.730 MeV
Eγ (intensity)	112.9 keV (6.17%)	25.7 keV (23.2%)	165.0 keV (26%)
	208.4 keV (10.4%)	48.9 keV (17.0%)	352.2 keV (29%)
		74.6 keV (10.2%)	388.6 keV (18%)
			652.1 keV (16%)

### *In vivo* combination therapy of pemetrexed and ^177^Lu-EC0800

**Figure 6:**
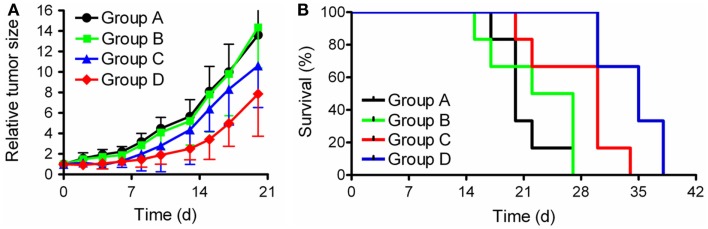
**Graphs of tumor growth and survival**. Preclinical study with athymic nude mice bearing KB tumor xenografts. **(A)** Relative tumor size and **(B)** survival rate of mice of group A (control), group B (2 × 0.8 mg pemetrexed), group C (20 MBq ^177^Lu-EC0800 and 0.4 mg pemetrexed), and group D (20 MBq ^177^Lu-EC0800 and 2 × 0.8 mg pemetrexed).

In a recent study the combined application of ^177^Lu-EC0800 (Figure 3A) and pemetrexed (Figure 1D) was investigated using KB tumor-bearing nude mice (79). Four groups of six athymic nude mice with KB tumor xenografts received only saline (group A), pemetrexed at a high dose (2 × 0.8 mg, group B), ^177^Lu-EC0800 (1 × 20 MBq), and pemetrexed at a low dose (1 × 0.4 mg) to reduce renal uptake (group C) or ^177^Lu-EC0800 (1 × 20 MBq) and a high dose of pemetrexed (2 × 0.8 mg, group D). Application of even a high dose of pemetrexed alone had almost no effect on tumor growth in mice of group B, compared with control animals of group A (Figure 6A). However, the application of ^177^Lu-EC0800 resulted in a tumor growth delay (group C) which was increased if it was combined with a high dose of pemetrexed (group D) (Figure 6A). The therapeutic effect of ^177^Lu-EC0800 was also reflected by the increased average survival time of mice (group C: 30 days) compared to control mice (group A: 20 days) and mice which received only pemetrexed (group B: 24.5 days) (Figure 6B) (79). However, the clearly most favorable therapy protocol was the combination of ^177^Lu-EC0800 with a high dose of pemetrexed (group D) which resulted in an average survival time of 35 days (Figure 6B) (79).

These findings indicated a radiosensitizing effect of pemetrexed as it was previously reported in other *in vitro* and *in vivo* studies that combined pemetrexed with external radiation therapy (72, 73, 80, 81). An additional study was conducted with non-tumor-bearing mice over a time period of several months to investigate the potential impairment of the kidneys. It was demonstrated that pemetrexed was able to protect kidneys from radio-nephrotoxicity (79). Further and more detailed studies will be warranted to investigate the utility of this combination in view of a potential clinical application.

### Folate receptor targeted radionuclide tumor therapy using ^177^Lu-cm09

**Figure 7:**
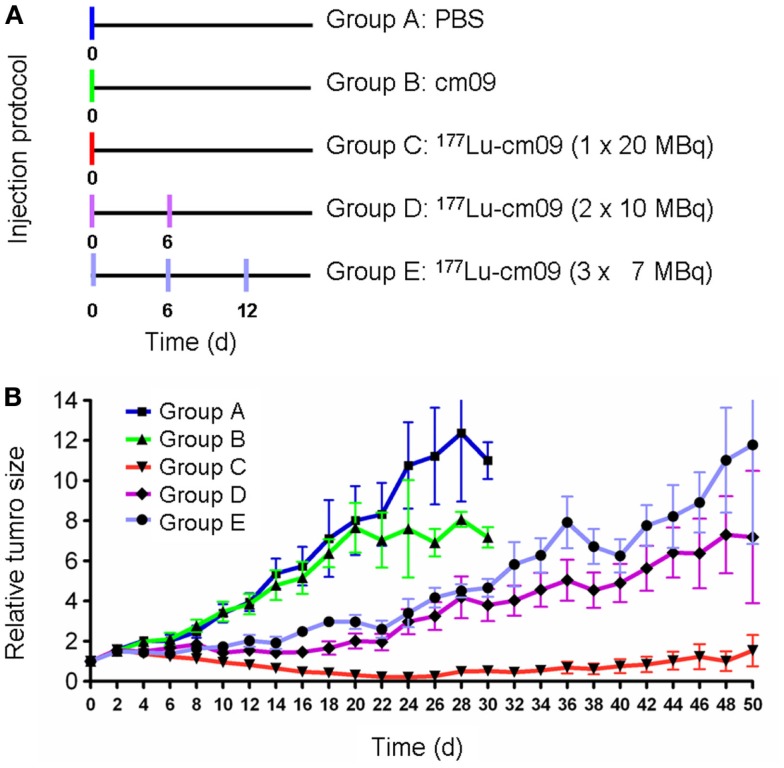
**Design and results of the therapy study**. **(A)** Application protocol and **(B)** average relative tumor size for mice of groups A–E. This research was originally published in Ref. (64). ©by the Society of Nuclear Medicine and Molecular Imaging, Inc.

The first published therapy experiment with a folate radioconjugate was performed with KB tumor-bearing nude mice using ^177^Lu-cm09 (64). Groups of five mice each were treated with only saline (group A) or with the unlabeled DOTA-folate cm09 (group B). One group received ^177^Lu-cm09 in a single injection of 20 MBq (group C), another group received two injections of 10 MBq each (group D) and the last group received three injections of 7 MBq each (group E) (Figure 7A). The individual body weight and tumor volume was measured every other day. The results showed a constant tumor growth in mice of groups A and B which did not receive radioactivity (Figure 7B). In all mice of groups C to E tumor growth was clearly delayed with the best effect achieved in mice of group C which had been injected with the whole amount of 20 MBq ^177^Lu-cm09 in one single injection (Figure 7B). The average survival time of control mice of group A and group B was 27 and 24 days, respectively, whereas an almost doubled survival time was observed for mice of groups D and E (48 and 46 days, respectively). In mice which received 20 MBq ^177^Lu-cm09 in one single injection the tumors disappeared completely in four of five mice and hence these four mice were still alive at the end of the study which prevented the determination of an average survival time (64).

### Folate receptor targeted α- and β-radionuclide tumor therapy using ^149/161^Tb-cm09

By using the same DOTA-folate conjugate (cm09, Figure 4B) as previously employed with ^177^Lu (64) a pilot therapy study was performed with therapeutic terbium radioisotopes (82). ^161^Tb decays by the emission of β-particles and provides similar decay properties to ^177^Lu (Table 1) (82, 83). ^149^Tb decays by emission of short-ranged α-particles with a half-life of 4.12 h (Table 1) (82). Since the availability of ^149^Tb was limiting in this study the experiments were performed with only a small number of mice bearing KB tumor xenografts.

**Figure 8:**
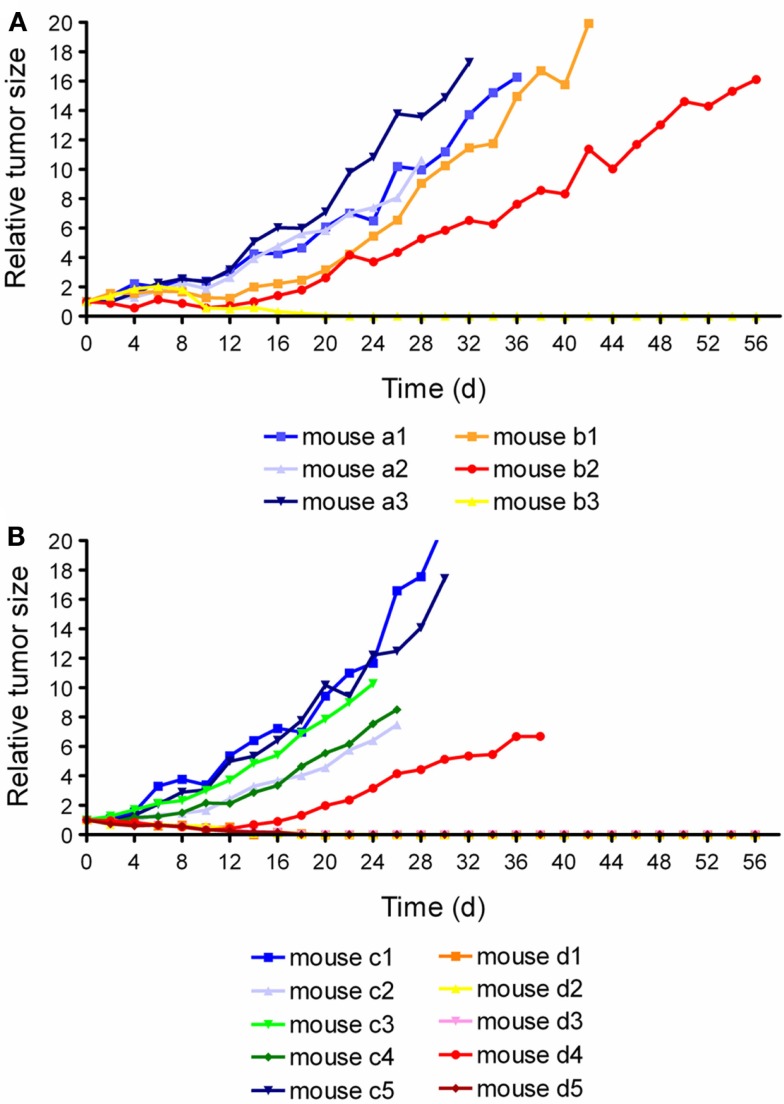
**Graphsof tumor growth from each individual mouse**. **(A)** Relative tumor size of mice of the α-radionuclide therapy study (a1–a3: control mice; b1–b3: mice treated with ^149^Tb-cm09). **(B)** Relative tumor size of mice of the β^−^- radionuclide therapy study (c1–c5: control mice; d1–d5: ^161^Tb-cm09 treated mice). This research was originally published in Ref. (82). ©by the Society of Nuclear Medicine and Molecular Imaging, Inc.

In the α-therapy study, three mice received only saline (group A) and three mice received twice an injection of 1.1 and 1.3 MBq of ^149^Tb-cm09, respectively (group B). In two of the treated mice the tumor growth was clearly delayed and in one mouse the tumors disappeared completely (Figure 8A). Compared to untreated control mice of group A, the average survival time was significantly prolonged (*p* < 0.05) in mice of group B. The β^−^-therapy study was carried out with five mice which received only saline (group A) and another five mice which received 11 MBq of ^161^Tb-cm09 (group B). The overall survival time of control mice was 28 days. In four of the five treated mice of group B, KB tumors disappeared completely and hence these mice were still alive at day 56 when the study was terminated (Figure 8B).

The results obtained with ^177^Lu-cm09 and ^149/161^Tb-cm09 indicated the potential of FR-targeted radionuclide therapy by using folic acid as a targeting ligand. Although the therapy regimes reported in these studies were well tolerated by the test animals, there is a potential risk of damage to the kidneys by particle-radiation. Therefore, kidney function was monitored in a preliminary study over 6 months in non-tumor-bearing mice which received ^177^Lu-cm09 (20 MBq/mouse) (unpublished data). Blood plasma parameters were analyzed and quantitative SPECT experiments using ^99m^Tc-DMSA were performed to estimate potential impairment of kidney function as previously reported (84). Although signs of radio-nephrotoxicity were not observed, more recent investigations indicate long-term impairment of the kidney function if higher amounts of folate radioconjugates are administered. Hence, further preclinical studies with larger cohorts of mice will be clearly necessary to provide more detailed information about the suitability of this therapy concept.

### Folate receptor targeted radionuclide tumor therapy using radioactive liposomes

In terms of radionuclide therapy the approach of using liposome- or nanoparticle-based carriers for passive or targeted delivery of particle-emitting radionuclides to cancer cells is scarcely reported in the literature but has recently been reviewed by Sofou (85). It could be in particular attractive for radionuclides that are inappropriate for direct complexation by conventional chelation concepts (86). An example that made use of folic acid decorated liposomes as carriers for the α-emitting radionuclides radium (^223^Ra) and actinium (^225^Ac) has been reported by Larsen and Co-workers (87). The radioactive liposome formulations possessed binding properties to FR-expressing cancer cells *in vitro* and were stable in serum with only low release of radionuclides. Hence, this or similar approaches may have the potential for an *in vivo* translation in the future.

### Stability, radiolysis, toxicity, and safety aspects of folate-based radioconjugates

Folate radioconjugates were found to be very stable in PBS and blood plasma at 37°C with only marginal release of radiometals over time (56). ^177^Lu-cm09 showed a particularly high *in vitro* stability with >99% intact product over 6 days in human plasma (64). Little is known about potential radiolysis of folate radioconjugates (64). However, since there are studies reporting on the degradation of folate molecules exposed to high temperatures, ultraviolet light, and oxygen (88–90) it is likely that radiolytic processes would occur in highly active formulations of radiofolates. In view of a clinical application of therapeutic folate radioconjugates extensive investigations will be required with respect to the stabilization of radioconjugates by using quenchers such as for instance ethanol and ascorbic acid.

Since folic acid and folates are essential nutritions for the human organism and can be administered by supplements in mg-amounts, folate derivatives are expected to be well tolerated even if they were applied for radiotherapeutic purposes, where the injected amount would not exceed about 200 μg. Nevertheless, the high uptake of folate radioconjugates in the kidneys is of concern in terms of long-term radionephropathy caused by particle-radiation if the folate conjugates were used for radionuclide therapy as mentioned above.

## Challenges for Clinical Translation and Future Perspectives

For a clinical translation of the “pemetrexed protocol” in combination with therapeutic radiofolates it will be crucial to answer the question on whether pemetrexed would have the same kidney reducing effect of radiofolates in man than it has in mice. Given that this would be the case a further critical point may be the fact that pemetrexed is not yet approved for ovarian, endometrial, and cervical cancer, which are the tumor types with the highest FR-expression level (6, 9). At the moment non-small cell lung cancer would clearly be the ideal candidate for this combination because of frequent FR-expression in this cancer type and the FDA-approved indication of pemetrexed for the treatment of non-small cell lung cancer (91–93). However, preclinical results of this combination in a lung tumor mouse model will be required to confirm the applicability of this approach also in other than cervical and ovarian tumors types.

The development of a folate conjugate which was chemically modified with an albumin-binding entity meant a significant step forward in the development of a folate radioconjugates for therapeutic applications (64). Excellent results were achieved with ^177^Lu-cm09 and ^149/161^Tb-cm09 in terms of tumor growth inhibition and tolerability in mice (64, 82). For a safe application of targeted radionuclide therapy the tumor-to-kidney ratio of accumulated radioactivity should be above one. In this respect, it has to be critically acknowledged that the uptake and retention of radiolabeled cm09 in the kidneys is still high relative to the tumor uptake. This aspect needs to be considered with regard to a potential clinical translation of this approach. Future development must clearly focus on alteration of the tracer design. Such an intervention is expected to result in an improved tissue distribution profile with respect to an increased tumor-to-kidney ratio of radioactivity.

## Summary and Conclusion

Targeted radionuclide therapy showed impressive results for the palliative treatment of cancer such as neuroendocrine tumors. Due to the large number of tumor types which overexpress the FR and the ideal characteristics of folic acid as a targeting ligand the development of therapeutic folate radioconjugates holds great promise for the management of cancer diseases in the future. The drawback of a high renal accumulation of conventional radiofolates can largely be overcome by co-application of the antifolate pemetrexed or by a chemical modification of the folate radioconjugate with an albumin-binding entity. Both strategies resulted in significantly increased tumor-to-kidney ratios and hence, allowed the performance of preclinical therapy studies using particle-emitting isotopes. Application of ^177^Lu-EC0800 benefited from the co-administered pemetrexed not only in that it reduced undesired kidney uptake but also by an enhanced anticancer effect which was mediated by pemetrexed’s action as a chemotherapeutic agent. Nevertheless, application of a chemically modified folate radioconjugate with optimized tissue distribution characteristics is more likely translatable to a clinical setting than the combination with a chemotherapeutic agent. We believe that a further improvement of the tracer design will finally allow application of FR-targeted radionuclide therapy in patients.

## Conflict of Interest Statement

The authors declare that the research was conducted in the absence of any commercial or financial relationships that could be construed as a potential conflict of interest.
